# Is the Combination of Plain X-ray and Probe-to-Bone Test Useful for Diagnosing Diabetic Foot Osteomyelitis? A Systematic Review and Meta-Analysis

**DOI:** 10.3390/jcm12165369

**Published:** 2023-08-18

**Authors:** María del Mar Calvo-Wright, Francisco Javier Álvaro-Afonso, Mateo López-Moral, Yolanda García-Álvarez, Esther García-Morales, José Luis Lázaro-Martínez

**Affiliations:** Diabetic Foot Unit, Clínica Universitaria de Podología, Facultad de Enfermería, Fisioterapia and Podología, Universidad Complutense de Madrid, Instituto de Investigación Sanitaria del Hospital Clínico San Carlos (IdISSC), 28040 Madrid, Spain; mcalvo10@ucm.es (M.d.M.C.-W.); matlopez@ucm.es (M.L.-M.); ygarci01@ucm.es (Y.G.-Á.); eagarcia@ucm.es (E.G.-M.); diabetes@ucm.es (J.L.L.-M.)

**Keywords:** diabetic foot, diabetic foot ulcer, diabetic foot osteomyelitis, probe-to-bone test, plain X-ray

## Abstract

A systematic review and meta-analysis was conducted to assess the diagnostic accuracy of the combination of plain X-ray and probe-to-bone (PTB) test for diagnosing diabetic foot osteomyelitis (DFO). This systematic review has been registered in PROSPERO (a prospective international register of systematic reviews; identification code CRD42023436757). A literature search was conducted for each test separately along with a third search for their combination. A total of 18 articles were found and divided into three groups for separate analysis and comparison. All selected studies were evaluated using STROBE guidelines to assess the quality of reporting for observational studies. Meta-DiSc software was used to analyze the collected data. Concerning the diagnostic accuracy variables for each case, the pooled sensitivity (SEN) was higher for the combination of PTB and plain X-ray [0.94 (PTB + X-ray) vs. 0.91 (PTB) vs. 0.76 (X-ray)], as was the diagnostic odds ratio (DOR) (82.212 (PTB + X-ray) vs. 57.444 (PTB) vs. 4.897 (X-ray)). The specificity (SPE) and positive likelihood ratio (LR+) were equally satisfactory for the diagnostic combination but somewhat lower than for PTB alone (SPE: 0.83 (PTB + X-ray) vs. 0.86 (PTB) vs. 0.76 (X-ray); LR+: 5.684 (PTB + X-ray) vs. 6.344 (PTB) vs. 1.969 (X-ray)). The combination of PTB and plain X-ray showed high diagnostic accuracy comparable to that of MRI and histopathology diagnosis (the gold standard), so it could be considered useful for the diagnosis of DFO. In addition, this diagnostic combination is accessible and inexpensive but requires training and experience to correctly interpret the results. Therefore, recommendations for this technique should be included in the context of specialized units with a high prevalence of DFO.

## 1. Introduction

Diabetic foot ulcers (DFUs) have been described as one of the most prevalent complications related to diabetes mellitus (DM) [1]. Approximately 50% of diabetic foot disease cases are at risk of developing a foot infection [2]. Diabetic foot infection (DFI) is the cause of almost 85% of foot amputations in people with DM and has been linked to an increase in morbidity, increased costs, and decreased quality of life [3,4]. DFIs can lead to osteomyelitis and spread contiguously to deeper tissues, including the bones [5]. Diabetic foot osteomyelitis (DFO) is a severe complication of diabetic foot disease and can affect 50–60% of severe DFI cases and approximately 20% of moderate DFIs [6,7].

Although bone histopathology and culture provide the standard criteria for diagnosing DFO [5], resources or expertise to perform bone biopsy are unavailable in many settings. The International Working Group on Diabetic Foot (IWGDF) recommends detecting DFO as early as possible to prevent further complications such as foot amputation and death [8]. Plain X-ray is the first imaging modality used for the diagnosis of DFO [8]. However, the classic radiological triad comprising osteolysis, periosteal reaction, and bone destruction is generally not evident until a later stage occurring at least 10 to 20 days after onset of symptoms. Imaging studies for diagnosing DFO have reported low sensitivity (SEN) of 43–75% and specificity (SPE) of 75–83% using conventional X-ray [9,10], especially in early infection [11].

The probe-to-bone (PTB) test is widely used for clinical outpatients to assess DFO and is performed with sterile metal forceps, such as Halsted mosquito forceps. The result is considered positive when the investigator can feel a sandy or hard surface [12]. The combination of PTB and X-ray tests has a SEN and SPE similar to those of other more expensive diagnostic tests such as magnetic resonance imaging (MRI) for the diagnosis of DFO (SEN 97% and SPE 92%) [13].

Studies have evaluated the performance characteristics of the PTB test in the diagnosis of DFO [12,14,15,16,17]. Nevertheless, new analyses are needed due to the recent publication of new studies [13,18]. To the best of our knowledge, no systematic reviews and meta-analyses have evaluated the diagnostic performance of the combination of PTB and plain X-ray tests in the diagnosis of DFO thus far. The primary aim of this systematic review and meta-analysis was to evaluate and estimate the performance characteristics of the PTB test together with conventional X-ray and to determine the pretest probability at which this combination is useful for diagnosing osteomyelitis.

## 2. Materials and Methods

### 2.1. Literature Search 

This study was performed according to the Preferred Reporting Items for Systematic Reviews and Meta-Analyses (PRISMA) guidelines [19] and has been registered in PROSPERO (a prospective international register of systematic reviews; identification code CRD42023436757). Two reviewers (M.M.C.W. and F.J.Á.A.) independently searched three electronic databases (PubMed, Medline, and Cochrane) for relevant studies on the diagnosis of osteomyelitis using the PTB test or plain X-ray, spanning from inception until May 15, 2023. An independent search was carried out for each test by the two reviewers. The words “Osteomyelitis”, “Probe-to-bone”, “Diagnosis”, and “Diabetic Foot” where used as search terms. These keywords were directly combined using the Boolean operator “AND” to form the following search strategies: probe-to-bone AND osteomyelitis AND diabetic foot and probe-to-bone AND diagnosis AND osteomyelitis AND diabetic foot. 

For the second search, the keywords used were “Osteomyelitis”, “Plain X-ray”, “Diagnosis”, and “Diabetic Foot”. These terms were combined using the Boolean operator “AND” to form the following search strategies: plain X-ray AND osteomyelitis AND diabetic foot and plain X-ray AND diagnosis AND osteomyelitis AND diabetic foot. 

### 2.2. Selection Requirements 

The inclusion criteria were (a) studies published in English, (b) patients with suspected DFO and a positive PTB test (for the first search), and (c) studies using prospective/retrospective case series, case-control, cross-sectional, cohort, or randomized clinical trial designs. The exclusion criteria were (a) animal trials, (b) articles including only diagnostic tests other than PTB or plain X-ray for DFO, (c) articles unrelated to DFO, and (d) articles from which it was not possible to extract the data required for the meta-analysis.

### 2.3. Literature Screening 

Following deduplication of search results, potential articles were reviewed based on the title and abstract. Articles were independently screened by two authors (M.M.C.W. and F.J.Á.A.), and the results were compared. Any disparity between the authors was resolved by a third reviewer (J.L.L.M.). The articles included in the systematic review were divided into three groups. The first was used for the validation of the PTB test, the second was used for the validation of plain X-ray, and the third was used for the validation of the combination of both tests.

### 2.4. Data Extraction

A customized Microsoft Excel spreadsheet was used to extract the data from the studies. A total of three spreadsheets were made, one for each group (PTB validation, plain X-ray validation, and combination test validation). The extracted data included the first author’s name, year of publication, study design, number of patients, evaluated test, comparative diagnostic test, and outcome measures (SEN, SPE, positive and negative predictive values (PPV and NPV), LR+ and negative likelihood ratio (LR−), and osteomyelitis prevalence). 

### 2.5. Quality Evaluation of Included Studies (STROBE Guidelines)

Three independent researchers analyzed the data collected from all the articles. Since the included articles were prospective, retrospective, and cross-sectional studies, the quality evaluation was based on the standard STROBE guidelines to help guarantee high-quality presentation of observational studies [20]. Reviewers evaluated the adequacy of reported items using the STROBE checklist. This checklist provides a framework to ensure completeness and transparency. 

The STROBE checklist has 22 items: item 1, title and abstract; items 2 and 3, introduction; items 4–12, methods; items 13–17, results; items 18–21, discussion; and item 22, funding and sponsorship. Two reviewers (M.M.C.W. and F.J.Á.A.) independently assessed each study using the STROBE guidelines. A third reviewer (J.L.L.M.) helped to achieve a consensus in cases of disagreement.

### 2.6. Statistical Analyses

The meta-analysis was carried out using a web application for meta-analysis of diagnostic test accuracy data Meta-DiSc version 2.2 (https://ciberisciii.shinyapps.io/MetaDiSc2/, accessed on 2 July 2023) [21] with a bivariate or univariate random-effects model. Pooled SEN and SPE were calculated for PTB, plain radiography, and their combination. The heterogeneity (I^2^), correlation index, LR+, and diagnostic odds ratio (DOR) were extracted for each test separately. The receiver operating characteristic (ROC) curve was also obtained, and the 95% confidence interval of the area under the curve (AUC 95%) was calculated. A bivariate random-effects model was used. A second statistical analysis was done for each test by extracting the studies that used histopathology as a reference, which is considered the gold standard for the diagnosis of osteomyelitis [5]. For the analysis of the combination of PTB + plain X-ray, LR+ and I^2^ for SEN and SPE were extracted. All the studies included in this analysis used histopathology as a comparative diagnostic test.

## 3. Results

### 3.1. Literature Retrieval

**Figure 1:**
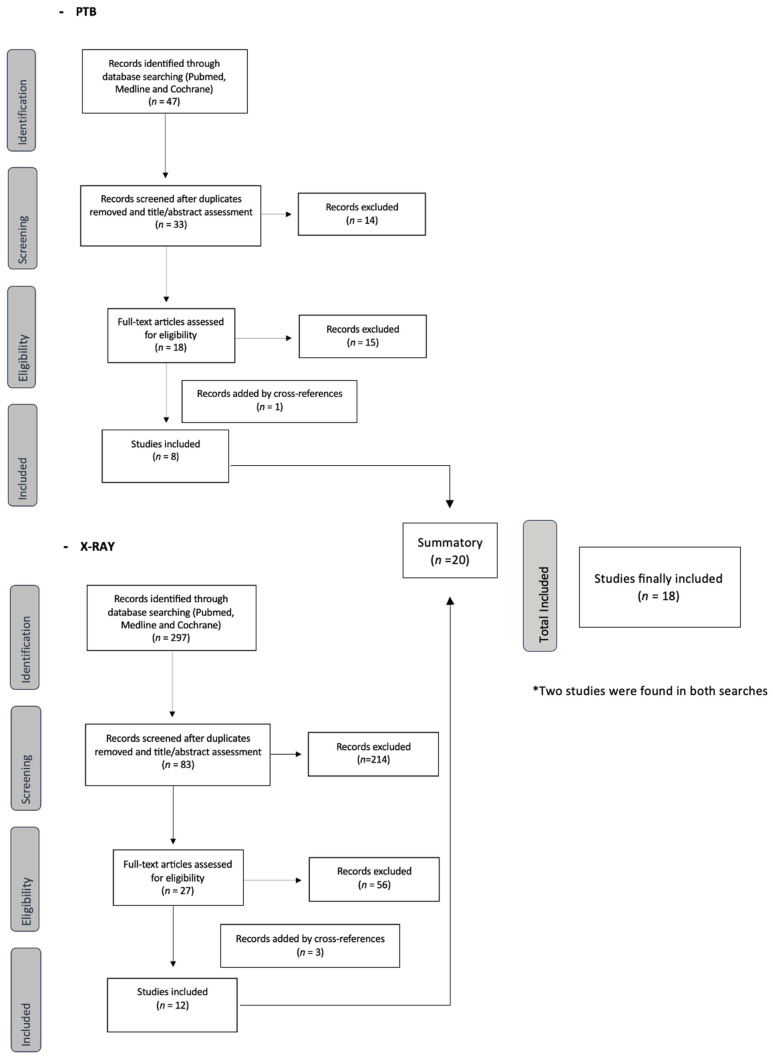
Flowchart of identified studies.

In a first search with the application of the inclusion criteria, 47 articles for PTB and 297 articles for plain X-ray were identified. After eliminating duplicates and reading the titles and abstracts, 18 articles for PTB and 27 articles for plain X-ray were selected for full-text evaluation. Finally, after eliminating the coinciding articles from the two searches, 18 studies were included in the analysis. Figure 1 shows the literature screening process. 

### 3.2. Quality of the Reporting

The most poorly completed items by the included studies were 9 (bias), 10 (study size), 13 (participants), and 21 (generalizability). Table 1 shows the overall rating for the STROBE checklist.

### 3.3. Outcome Measures

#### 3.3.1. PTB

In order to determine the accuracy of this test, pooled SEN and SPE were extracted using a random-effects model, which showed a mean SEN of 0.84 and a mean SPE of 0.82 for all included studies [12,13,14,15,16,17,22,23]. High heterogeneity was found in this analysis with an I^2^ value of 81.9%. The correlation index was low with an estimate of −0.127, and the LR+ estimate was 4.77. The DOR was 24.125 [12,13,14,15,16,17,22,23], and AUC 95% was 0.736.

**Figure 2:**
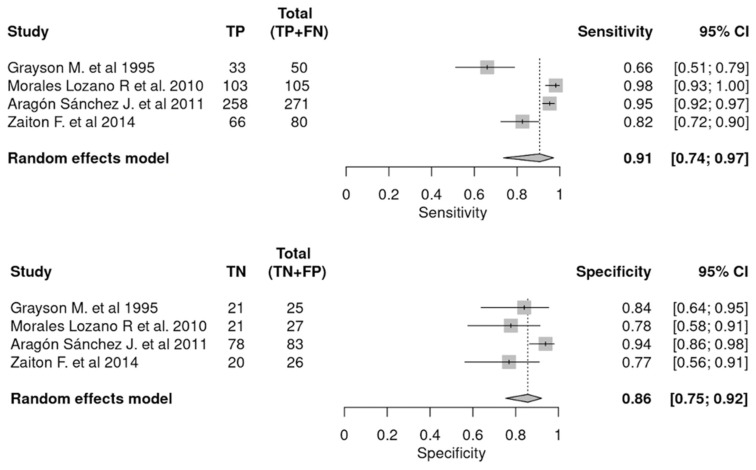
PTB’s pooled sensitivity and specificity compared to histopathology [12,13,16,23]. *TP, true positive; FN, false negative; TN, true negative; FP, false positive; CI, confidence interval*.

**Figure 3:**
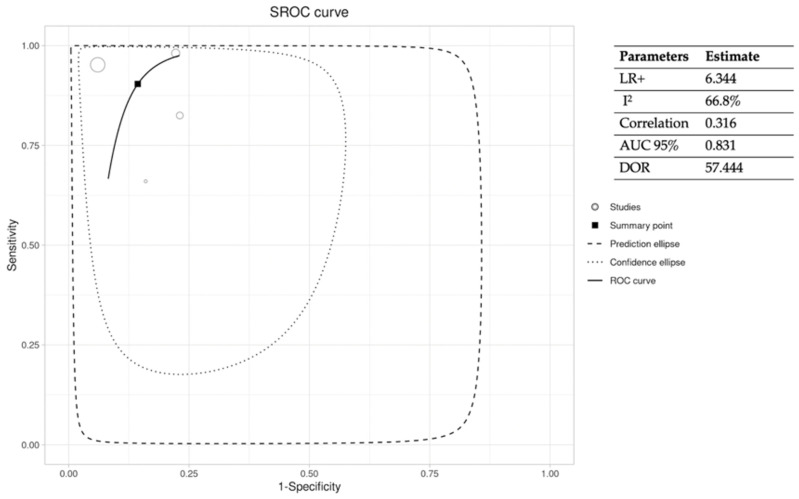
ROC curve and extracted statistical variables of PTB–Histopathology studies. *LR+, positive likelihood ratio; I^2^, heterogeneity; AUC 95%, 95% confidence interval of the area under the curve; DOR, diagnostic odds ratio*.

These same variables were extracted in a sub-analysis of the articles comparing the PTB test with histopathology (gold standard) [12,13,14,16]. The results are shown in Figure 2 and Figure 3. Figure 2 shows the pooled SEN and SPE of the PTB test compared to histopathology. Figure 3 shows the ROC curve analysis and estimates of LR+, I^2^, correlation, DOR, and AUC 95% of the studies selected.

Related to the sample size, there were a total of 1156 participants in the articles included in this group [12,13,14,15,16,17,22,23], of which 550 were considered true positives for DFO diagnosed by PTB. Considering only the articles that compared the results of PTB to gold standard, the sample was 667, of which 460 were true positives. These data are shown broken down by article in Figure 2.

#### 3.3.2. Plain X-ray

In the first analysis including all the studies [24,25,26,27,28,29,30,31,32,33], the pooled SEN and SPE were 0.68 and 0.74, respectively. I^2^ was estimated as 61.3%, LR+ was 2.61, the correlation index was −0.643, and DOR was 6.116. In the ROC curve analysis, AUC 95% was 0.496.

**Figure 4:**
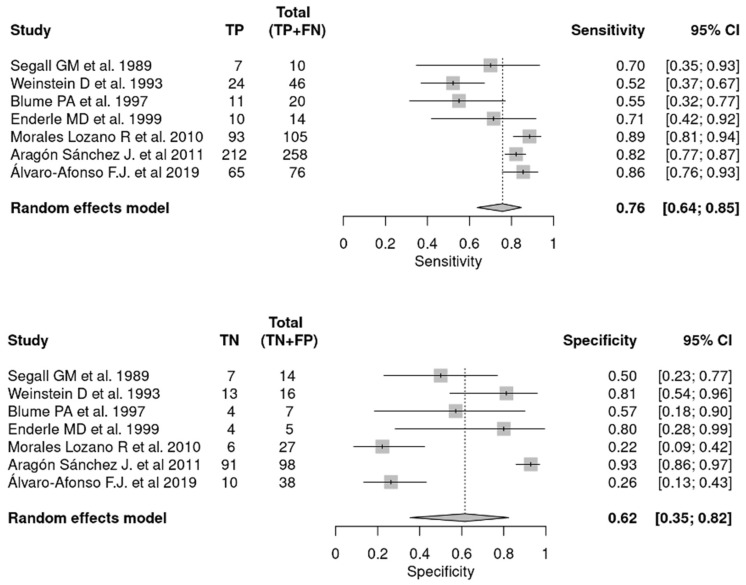
Plain X-ray’s pooled sensitivity and specificity compared to histopathology [13,16,25,27,30,31,33]. *TP, true positive; FN, false negative; TN, true negative; FP, false positive; CI, confidence interval*.

**Figure 5:**
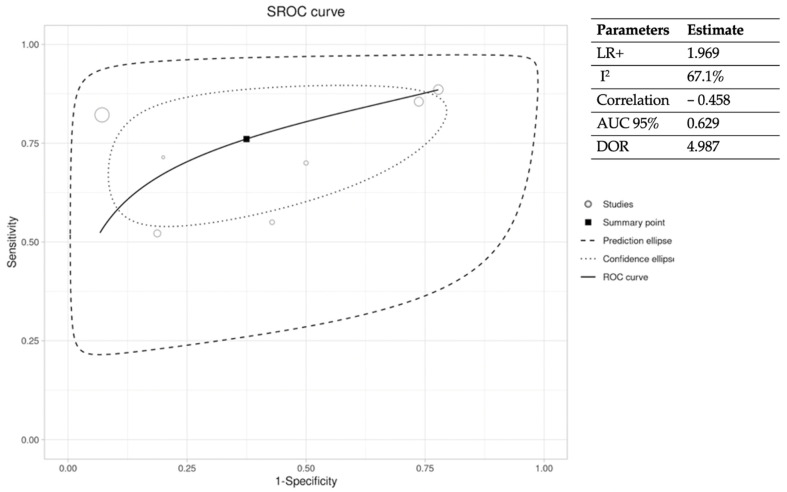
ROC curve and extracted statistical variables of plain X-ray and histopathology studies. *LR+, positive likelihood ratio; I^2^, heterogeneity; AUC 95%, 95% confidence interval of the area under the curve; DOR, diagnostic odds ratio*.

When comparing the accuracy of plain X-ray with histopathology as the gold standard [13,16,25,27,30,31,33], changes were found in the parameters evaluated, which are shown in Figure 4 and Figure 5. Figure 4 shows the pooled SEN and SPE of plain X-ray compared to histopathology (gold standard). Figure 5 shows the ROC curve analysis and the estimates of LR+, I^2^, correlation, DOR, and AUC 95% of the studies selected. There was a total sample of 963 in the articles included in this group [24,25,26,27,28,29,30,31,32,33], of which 496 were true positives for DFO diagnosed by plain X-ray.

The sample size for the subgroup of studies that compared X-ray to histopathology was 734, of which 422 were true positives. These data are shown broken down by article in Figure 4.

#### 3.3.3. Combination of PTB and Plain X-ray

**Figure 6:**
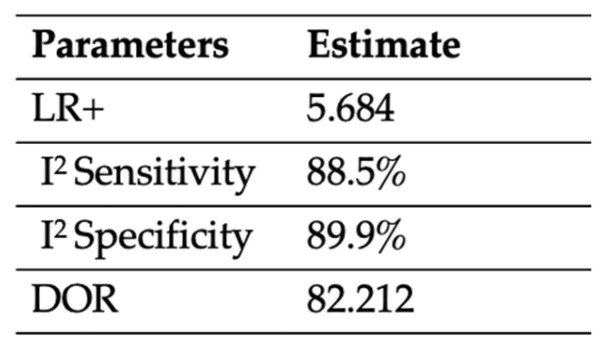
Extracted statistical variables of the combination of PTB + plain X-ray. *LR+, positive likelihood ratio; I^2^ SEN, sensitivity heterogeneity; I^2^ SPE, specificity heterogeneity; DOR, diagnostic odds ratio*.

**Figure 7:**
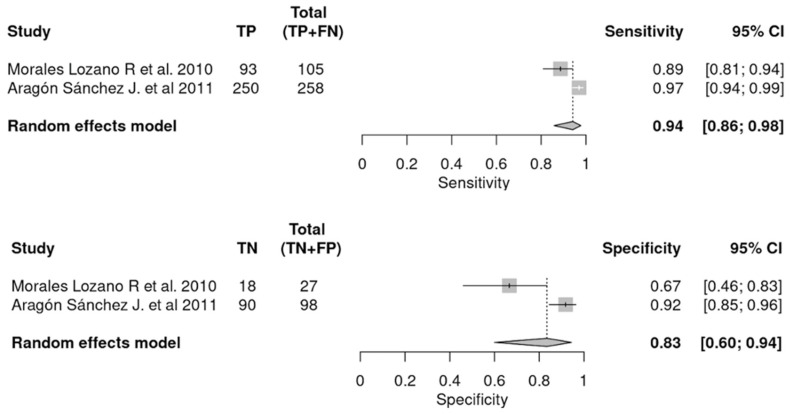
Combined tests’ sensitivity and specificity pools compared to histopathology [13,16]. *TP, true positive; FN, false negative; TN, true negative; FP, false positive; CI, confidence interval*.

Two studies analyzed the diagnostic combination of PTB + plain X-ray compared to the gold standard [13,16]. In this case, a univariate analysis was carried out in order to determine the diagnostic accuracy of the combination of these tests. From the statistical analysis, the pooled SEN was 0.94, the heterogeneity was 88.5%, the SPE was 0.83, and the relative heterogeneity was 89.9%. LR+ was 5.874, and DOR was 82.212. These data are shown in Figure 6 and Figure 7. Figure 6 shows the estimates of LR+, I^2^ SEN, I^2^ SPE, and DOR. Figure 7 shows the pooled SEN and SPE of the combination of PTB and plain X-ray.

The total sample of patients included in the two articles assessing the diagnostic combination [13,16] was 488, of which 343 were true positives. These data are shown broken down by item in Figure 7.

## 4. Discussion

Based on the data obtained in this systematic review and meta-analysis, it can be determined that the combination of PTB and plain X-ray demonstrates high diagnostic accuracy for DFO. This diagnostic combination shows a SEN of 0.94, which means that out of every 100 patients with a diagnosis of DFO (established by histopathology as the gold standard), 94 are correctly diagnosed (true positives). This high SEN makes it highly unlikely for a patient to have DFO if the test results are negative. When the tests were analyzed separately, the mean SEN of the studies was lower at 0.91 for PTB and 0.76 for plain X-ray. These values were even lower when the analysis included studies that did not use the gold standard as a reference test for the diagnosis of DFO.

SPE represents the ability to determine that a negative test result actually corresponds to a patient without DFO (true negative). The pooled value was 0.83 for the diagnostic combination of PTB + plain X-ray. This value is much higher than that obtained in the plain radiographic analysis (0.76) and slightly lower than that shown by the PTB test alone (0.86). It is well known that as a test becomes more sensitive, it becomes somewhat less specific, so we can determine that the values obtained show good diagnostic accuracy for the test combination.

DOR represents the effectiveness of a diagnostic test and was 82.212 for the diagnostic combination of PTB and plain X-ray. For each test separately, lower values were obtained at 57.444 for PTB and 4.897 for plain X-ray. For this variable, values above 1 indicate discriminatory capacity, which is greater when the DOR is higher. Thus, the obtained DOR value can be considered high for the diagnostic combination of PTB + plain X-ray, indicating that it is effective for the diagnosis of DFO.

LR+ represents how much more likely it is that a patient would have a disease (DFO in this case) after obtaining a positive test result and is independent of prevalence. For plain X-ray, the LR+ was 1.969, which is considered bad. For analyses of PTB alone and the combination of PTB + plain X-ray, the LR+ values were 6.344 and 5.684, respectively, which are considered good and can be extrapolated to populations with other prevalence rates of osteomyelitis.

The most recent systematic review and meta-analysis on the diagnostic accuracy of imaging tests for DFO [34] that included X-ray performance was published in 2020. That study showed that among all the imaging tests evaluated, MRI was the most accurate with SEN 96.4% and SPE 83.8%. These values are much higher than those obtained for plain X-ray but are similar to that obtained from the diagnostic combination of PTB + plain X-ray (SEN 0.94, SPE0.83) and can be compared to histopathological analysis (the gold standard). Both MRI and histopathological analysis are costly tests and may not be readily available. Therefore, the diagnostic combination of PTB + plain X-ray could result in a more accessible and cost-effective option for the diagnosis of DFO. However, it is currently necessary to carry out a cost-effectiveness studies of each of the diagnostic tests.

The reproducibility of PTB, plain X-ray, and the combination of both diagnostic tests has been assessed in several studies. García-Morales et al. [35] showed that the inter-observer variability of PTB in the diagnosis of DFO was statistically significant depending on the experience of the clinician. The PTB test demonstrated moderate to fair concordance with an experienced examiner, but the degree of concordance was not significant between a very experienced professional, a medium-experienced professional, and a healthcare professional without experience in diabetic foot.

Álvaro-Afonso et al. [36] performed a study to assess the influence of the location of the ulcer on the interpretation of the PTB test. They observed a stronger association between the results from clinicians with different levels of experience for ulcers located in the hallux and in the central metatarsals. There was poorer agreement for ulcers located in the lesser toes.

Another study analyzed the inter-observer and intra-observer variability in plain radiography in the diagnosis of DFO [37]. It was found that when using only plain radiography, low concordance rates were observed for clinicians with a similar level of experience. Intra-observer agreement was highest among experienced clinicians, followed by moderately experienced clinicians and inexperienced clinicians. This shows that using plain radiography for the diagnosis of DFO is dependent on the operator and shows low association strength, even among experienced clinicians, when interpreted in isolation without knowing the clinical characteristics of the lesion.

Álvaro-Afonso et al. [38] later analyzed the inter-observer reproducibility of a sequential combination of the PTB test and X-ray in the diagnosis of DFO among experienced clinicians. They observed very good agreement in the interpretation of the PTB test and good agreement in the interpretation of radiographs for the diagnosis of DFO. Based on these results, the authors consider that the interpretation of radiography will be easier if the clinician explores the ulcer beforehand or at least receives clinical information about it. This will make the final diagnosis more reliable. This also demonstrates the importance of jointly considering clinical information (PTB test) and diagnostic tests (simple radiography) to increase agreement among clinicians in the diagnosis of DFO. All these reproducibility studies show that a lack of agreement among professionals with similar or different levels of experience can lead to different diagnostic approaches and therapies that may sometimes be inadequate. Thus, there is a need to implement training programs for these diagnostic tests when establishing specialist diabetic foot units.

Our review has several limitations. First, we would like to point out that the literature is scarce, so no exclusion criteria have been applied with respect to the year of publication, which has meant the inclusion of numerous articles published more than 20 years ago. The two studies on the diagnostic combination of PTB and plain X-ray are more recent, but more and new studies on this subject are needed to provide more reliable results.

With regard to statistical analysis, an ROC curve analysis of the diagnostic combination could not be performed due to the number of articles included. There were only two articles that assessed the diagnostic accuracy of this combination [13,16], so the results should be interpreted with caution. The results may be extrapolated to centers with similar prevalence of osteomyelitis (>70%) that include professionals who are trained in this field.

It should be noted that the limitations of this review are largely a consequence of the limitations in the identified studies. There were numerous concerns about the potential for bias in the included studies. As shown in Table 1, methods to reduce the risk of bias and select patients were not completed in most of the studies included in this review. The most poorly completed items by the included studies were 9 (bias), 10 (study size), 13 (participants), and 21 (generalizability) based on the STROBE checklist.

The differences in the prevalence of DFO and the type of lesions included in the different studies could explain the high heterogeneity obtained in the meta-analyses. Studies conducted in specialized units within a hospital setting [13] showed a higher prevalence of DFO and lesions with more severe or acute infectious conditions than those conducted in an outpatient setting [16,33]. We found studies with DFO prevalence >75% [13,16,23], studies with prevalence of 49–75% [12,17,22,24,27,30,31,33], and studies with lower prevalence of 12–34% [14,15,26,29,33]. However, previous systematic reviews and meta-analyses [9,34,39] have analyzed the performance of each test separately, but not the combination of both tests, which is one of the strengths of our study. Another strength of this study is the analysis of studies that used histopathology as a reference diagnostic test [12,13,16,23,25,27,30,31,33], which provides more reliable and accurate results, as well as a more homogeneous analysis.

## 5. Conclusions

The combination of PTB and plain X-ray could be considered useful for the diagnosis of DFO as it shows high diagnostic accuracy comparable to that of MRI and histopathology diagnosis (the gold standard). This diagnostic combination is accessible and inexpensive but requires training and experience to correctly interpret the results. Therefore, recommendations for this combination should be included in the context of specialized units with a high prevalence of DFO. Diabetic foot healthcare professionals should be trained in the performance and interpretation of these diagnostic tests so that they can be included in the day-to-day clinical practice and promote early diagnosis to prevent consequences of DFO. However, it should be noted that the literature is sparse, and more studies are needed to support these findings with more evidence.

## Figures and Tables

**Table 1:** Overall rating for Strengthening the Reporting of Observational studies in Epidemiology (STROBE).

Item Number–STROBE Guidelines																							
	1 (a)	1 (b)	2	3	4	5	6	7	8	9	10	11	12	13	14	15	16	17	18	19	20	21	22
Grayson M. 1995, [12]	Yes	Yes	Yes	Yes	Yes	Yes	Yes	Yes	Yes	No	No	No	Yes	No	Yes	Yes	Yes	Yes	Yes	Yes	Yes	No	Yes
Shone A. 2006, [14]	Yes	No	No	No	No	Yes	No	Yes	No	No	No	No	No	Yes	Yes	Yes	No	Yes	Yes	No	No	Yes	Yes
Lavery LA. 2007, [15]	Yes	Yes	Yes	Yes	Yes	Yes	Yes	Yes	Yes	No	Yes	Yes	Yes	Yes	Yes	Yes	Yes	Yes	Yes	Yes	Yes	Yes	Yes
Morales Lozano R. 2010, [16]	Yes	Yes	Yes	Yes	Yes	Yes	Yes	No	Yes	No	Yes	Yes	Yes	No	Yes	Yes	Yes	Yes	Yes	Yes	Yes	No	Yes
Aragón Sánchez J. 2011, [13]	Yes	Yes	Yes	No	Yes	Yes	Yes	Yes	Yes	No	No	Yes	Yes	Yes	No	Yes	Yes	No	Yes	Yes	Yes	Yes	Yes
Mutluoglu M. 2012, [17]	Yes	Yes	Yes	Yes	No	Yes	Yes	Yes	Yes	No	No	Yes	Yes	Yes	Yes	No	Yes	Yes	Yes	Yes	Yes	No	Yes
Malone M. 2013, [22]	No	Yes	Yes	Yes	Yes	Yes	No	Yes	Yes	No	No	No	Yes	No	Yes	Yes	Yes	Yes	Yes	No	No	Yes	Yes
Zaiton F. 2014, [23]	Yes	Yes	Yes	Yes	Yes	Yes	Yes	Yes	Yes	No	No	Yes	Yes	Yes	No	No	Yes	Yes	Yes	Yes	Yes	No	Yes
Yuh WT. 1989, [24]	No	Yes	No	Yes	No	Yes	No	Yes	Yes	No	No	Yes	No	No	Yes	Yes	No	Yes	Yes	Yes	Yes	Yes	Yes
Segall GM. 1989, [25]	No	Yes	Yes	Yes	Yes	Yes	No	Yes	No	No	No	Yes	No	No	Yes	Yes	Yes	Yes	Yes	Yes	Yes	No	Yes
Largos G 1991, [26]	Yes	Yes	Yes	Yes	No	Yes	Yes	Yes	No	No	Yes	Yes	No	Yes	Yes	Yes	Yes	Yes	Yes	Yes	Yes	Yes	Yes
Weinstein D. 1993, [27]	Yes	Yes	Yes	Yes	Yes	Yes	Yes	Yes	Yes	No	No	No	Yes	Yes	Yes	No	Yes	No	Yes	Yes	Yes	No	No
Levine SE. 1994, [28]	No	Yes	Yes	No	Yes	No	No	Yes	Yes	No	No	Yes	Yes	No	Yes	Yes	Yes	Yes	Yes	No	Yes	No	Yes
Croll SD. 1996, [29]	Yes	Yes	Yes	Yes	No	Yes	Yes	Yes	No	No	No	Yes	Yes	No	Yes	Yes	Yes	No	Yes	Yes	Yes	No	Yes
Blume PA. 1997, [30]	Yes	Yes	Yes	Yes	Yes	Yes	No	Yes	Yes	No	No	Yes	Yes	No	Yes	Yes	Yes	Yes	Yes	No	No	Yes	Yes
Enderle MD. 1999, [31]	Yes	Yes	Yes	Yes	No	Yes	Yes	Yes	Yes	No	Yes	No	Yes	Yes	Yes	Yes	Yes	Yes	Yes	Yes	Yes	Yes	Yes
Nawaz A. 2009, [32]	No	Yes	Yes	Yes	Yes	Yes	Yes	Yes	No	No	Yes	Yes	No	No	Yes	No	Yes	Yes	Yes	Yes	Yes	Yes	Yes
Álvaro Afonso F.J. 2019, [33]	No	Yes	Yes	Yes	Yes	Yes	Yes	Yes	No	No	Yes	Yes	Yes	No	Yes	Yes	Yes	Yes	Yes	Yes	Yes	Yes	Yes

Red when it does not meet the criteria and green when it does. 1 (a), title; 1 (b), abstract.

## Data Availability

The data are available previous request to the corresponding author.

## References

[1] Lazzarini P.A., Pacella R.E., Armstrong D.G., Van Netten J.J. (2018). Diabetes-related lower-extremity complications are a leading cause of the global burden of disability. Diabet. Med..

[2] Prompers L., Huijberts M., Apelqvist J., Jude E., Piaggesi A., Bakker K., Edmonds M., Holstein P., Jirkovska A., Mauricio D. (2007). High prevalence of ischaemia, infection and serious comorbidity in patients with diabetic foot disease in Europe. Baseline results from the Eurodiale study. Diabetologia.

[3] Raspovic K.M., Wukich D.K. (2014). Self-reported quality of life in patients with diabetes: A comparison of patients with and without Charcot neuroarthropathy. Foot Ankle Int..

[4] Lázaro Martínez J.L., García Álvarez Y., Tardáguila-García A., García Morales E. (2019). Optimal management of diabetic foot osteomyelitis: Challenges and solutions. Diabetes Metab. Syndr. Obes..

[5] Lipsky B.A., Berendt A.R., Cornia P.B., Pile J.C., Peters E.J., Armstrong D.G., Deery H.G., Embil J.M., Joseph W., Karchmer A. (2012). 2012 infectious diseases society of America clinical practice guideline for the diagnosis and treatment of diabetic foot infections. Clin. Infect. Dis..

[6] Shahbazian H., Yazdanpanah L., Latifi S.M. (2013). Risk assessment of patients with diabetes for foot ulcers according to risk classification consensus of International Working Group on Diabetic Foot (IWGDF). Pak. J. Med. Sci..

[7] Lipsky B.A. (2008). Bone of contention: Diagnosing diabetic foot osteomyelitis. Clin. Infect. Dis..

[8] Lipsky B.A., Senneville É., Abbas Z.G., Aragón-Sánchez J., Diggle M., Embil J.M., Kono S., Lavery L.A., Malone M., Van Asten S.A. (2020). Guidelines on the diagnosis and treatment of foot infection in persons with diabetes (IWGDF 2019 update). Diabetes Metab. Res. Rev..

[9] Dinh M.T., Abad C.L., Safdar N. (2008). Diagnostic accuracy of the physical examination and imaging tests for osteomyelitis underlying diabetic foot ulcers: Meta-analysis. Clin. Infect. Dis..

[10] Butalia S., Palda V.A., Sargeant R.J., Detsky A.S., Mourad O. (2008). Does this patient with diabetes have osteomyelitis of the lower extremity?. JAMA.

[11] Berendt A.R., Peters E.J., Bakker K., Embil J.M., Eneroth M., Hinchliffe R.J., Jeffcoate W.J., Lipsky B.A., Senneville E., Teh J. (2008). Diabetic foot osteomyelitis: A progress report on diagnosis and a systematic review of treatment. Diabetes Metab. Res. Rev..

[12] Grayson M.L., Gibbons G.W., Balogh K., Levin E., Karchmer A.W. (1995). Probing to bone in infected pedal ulcers. A clinical sign of underlying osteomyelitis in diabetic patients. JAMA.

[13] Aragon-Sanchez J., Lipsky B.A., Lazaro-Martinez J.L. (2011). Diagnosing diabetic foot osteomyelitis: Is the combination of probe-to-bone test and plain radiography sufficient for high-risk inpatients?. Diabet. Med..

[14] Shone A., Burnside J., Chipchase S., Game F., Jeffcoate W. (2006). Probing the validity of the probe-to-bone test in the diagnosis of osteomyelitis of the foot in diabetes. Diabetes Care.

[15] Lavery L.A., Armstrong D.G., Peters E.J., Lipsky B.A. (2007). Probe-to-bone test for diagnosing diabetic foot osteomyelitis: Reliable or relic?. Diabetes Care.

[16] Morales Lozano R., González Fernández M.L., Martinez Hernández D., Beneit Montesinos J.V., Guisado Jiménez S., Gonzalez Jurado M.A. (2010). Validating the probe-to-bone test and other tests for diagnosing chronic osteomyelitis in the diabetic foot. Diabetes Care.

[17] Mutluoglu M., Uzun G., Sildiroglu O., Turhan V., Mutlu H., Yildiz S. (2012). Performance of the probe-to-bone test in a population suspected of having osteomyelitis of the foot in diabetes. J. Am. Podiatr. Med. Assoc..

[18] Meyr A.J., Seo K., Khurana J.S., Choksi R., Chakraborty B. (2018). Level of Agreement with a Multi-Test Approach to the Diagnosis of Diabetic Foot Osteomyelitis. J. Foot Ankle Surg..

[19] Tricco A.C., Lillie E., Zarin W., O’Brien K.K., Colquhoun H., Levac D., Moher D., Peters M.D.J., Horsley T., Weeks L. (2018). PRISMA Extension for Scoping Reviews (PRISMA-ScR): Checklist and Explanation. Ann. Intern. Med..

[20] Cuschieri S. (2019). The STROBE guidelines. Saudi J. Anaesth..

[21] Zamora J., Abraira V., Muriel A., Khan K.S., Coomarasamy A. (2006). Meta-DiSc: A software for meta-analysis of test accuracy data. BMC Med. Res. Methodol..

[22] Malone M., Bowling F.L., Gannass A., Jude E.B., Boulton A.J. (2013). Deep wound cultures correlate well with bone biopsy culture in diabetic foot osteomyelitis. Diabetes Metab. Res. Rev..

[23] Zaiton F., Samir A.M., Elkamash T.H., Tawfik A.M., Hadhoud K.M. (2014). Evaluation of diabetic foot osteomyelitis using probe to bone test and magnetic resonance imaging and their impact on surgical intervention. Egypt. J. Radiol. Nucl. Med..

[24] Yuh W.T., Corson J.D., Baraniewski H.M., Rezai K., Shamma A.R., Kathol M.H., Sato Y., El-Khoury G.Y., Hawes D.R., Platz C.E. (1989). Osteomyelitis of the foot in diabetic patients: Evaluation with plain film, 99mTc-MDP bone scintigraphy, and MR imaging. AJR Am. J. Roentgenol..

[25] Segall G.M., Nino-Murcia M., Jacobs T., Chang K. (1989). The role of bone scan and radiography in the diagnostic evaluation of suspected pedal osteomyelitis. Clin. Nucl. Med..

[26] Larcos G., Brown M.L., Sutton R.T. (1991). Diagnosis of osteomyelitis of the foot in diabetic patients: Value of 111In-leukocyte scintigraphy. AJR Am. J. Roentgenol..

[27] Weinstein D., Wang A., Chambers R., Stewart C.A., Motz H.A. (1993). Evaluation of magnetic resonance imaging in the diagnosis of osteomyelitis in diabetic foot infections. Foot Ankle.

[28] Levine S.E., Neagle C.E., Esterhai J.L., Wright D.G., Dalinka M.K. (1994). Magnetic resonance imaging for the diagnosis of osteomyelitis in the diabetic patient with a foot ulcer. Foot Ankle Int..

[29] Croll S.D., Nicholas G.G., Osborne M.A., Wasser T.E., Jones S. (1996). Role of magnetic resonance imaging in the diagnosis of osteomyelitis in diabetic foot infections. J. Vasc. Surg..

[30] Blume P.A., Dey H.M., Daley L.J., Arrighi J.A., Soufer R., Gorecki G.A. (1997). Diagnosis of pedal osteomyelitis with Tc-99m HMPAO labeled leukocytes. J. Foot Ankle Surg..

[31] Enderle M.D., Coerper S., Schweizer H.P., Kopp A.E., Thelen M.H., Meisner C., Pressler H., Becker H.D., Claussen C., Häring H.U. (1999). Correlation of imaging techniques to histopathology in patients with diabetic foot syndrome and clinical suspicion of chronic osteomyelitis. The role of high-resolution ultrasound. Diabetes Care.

[32] Nawaz A., Torigian D.A., Siegelman E.S., Basu S., Chryssikos T., Alavi A. (2010). Diagnostic performance of FDG-PET, MRI, and plain film radiography (PFR) for the diagnosis of osteomyelitis in the diabetic foot. Mol. Imaging Biol..

[33] Álvaro-Afonso F.J., Lázaro-Martínez J.L., García-Morales E., García-Álvarez Y., Sanz-Corbalán I., Molines-Barroso R.J. (2019). Cortical disruption is the most reliable and accurate plain radiographic sign in the diagnosis of diabetic foot osteomyelitis. Diabet. Med..

[34] Llewellyn A., Kraft J., Holton C., Harden M., Simmonds M. (2020). Imaging for detection of osteomyelitis in people with diabetic foot ulcers: A systematic review and meta-analysis. Eur. J. Radiol..

[35] García Morales E., Lázaro-Martínez J.L., Aragón-Sánchez F.J., Cecilia-Matilla A., Beneit-Montesinos J.V., González Jurado M.A. (2011). Inter-observer reproducibility of probing to bone in the diagnosis of diabetic foot osteomyelitis. Diabet. Med..

[36] Álvaro-Afonso F.J., Lázaro-Martínez J.L., Aragón-Sánchez F.J., García-Morales E., Carabantes-Alarcón D., Molines-Barroso R.J. (2014). Does the location of the ulcer affect the interpretation of the probe-to-bone test in the diagnosis of osteomyelitis in diabetic foot ulcers?. Diabet. Med..

[37] Álvaro-Afonso F.J., Lázaro-Martínez J.L., Aragón-Sánchez J., García-Morales E., Cecilia-Matilla A., Beneit-Montesinos J.V. (2013). Interobserver and intraobserver reproducibility of plain X-rays in the diagnosis of diabetic foot osteomyelitis. Int. J. Low. Extrem. Wounds.

[38] Álvaro-Afonso F.J., Lázaro-Martínez J.L., Aragón-Sánchez J., García-Morales E., García-Álvarez Y., Molines-Barroso R.J. (2014). Inter-observer reproducibility of diagnosis of diabetic foot osteomyelitis based on a combination of probe-to-bone test and simple radiography. Diabetes Res. Clin. Pract..

[39] Lam K., Van Asten S.A., Nguyen T., La Fontaine J., Lavery L.A. (2016). Diagnostic Accuracy of Probe to Bone to Detect Osteomyelitis in the Diabetic Foot: A Systematic Review. Clin. Infect. Dis..

